# Albertson (*Alb*) spectral radii and Albertson (*Alb*) energies of graph operation

**DOI:** 10.3389/fchem.2023.1267291

**Published:** 2023-09-29

**Authors:** Muhammad Mobeen Munir, Urwah Tul Wusqa

**Affiliations:** Department of Mathematics, University of the Punjab, Lahore, Pakistan

**Keywords:** Albertson (*Alb*) spectral radius, Albertson (*Alb*) energy, splitting graph, shadow graph, eigenvalues

## Abstract

The sum of the absolute eigenvalues of the adjacency matrix make up graph energy. The greatest absolute eigenvalue of the adjacency matrix is represented by the spectral radius of the graph. Both molecular computing and computer science have uses for graph energies and spectral radii. The Albertson (*Alb*) energies and spectral radii of generalized splitting and shadow graphs constructed on any regular graph is the main focus of this study. The only thing that may be disputed is the comparison of the (*Alb*) energies and (*Alb*) spectral radii of the newly formed graphs to those of the base graph. By concentrating on splitting and shadow graph, we compute new correlations between the *Alb* energies and spectral radius of the new graph and the prior graph.

## 1 Introduction

Let *G* be a simple undirected connected regular graph with the vertex set *V*(*G*) = {*v*_1_, *v*_2_, … , *v*_*z*_} and edge set *E*(*G*). If *v*_*k*_ and *v*_*l*_ are two nearby vertices of graph G, then *v*_*k*_ *v*_*l*_ is used to refer to the edge that connects them. We conventionally writes the vertex degree *d*_*k*_ related to the vertex *v*_*k*_ ∈ *V*(*G*) that represent total counting of edges end at a vertex *v*_*k*_ of a graph. The adjacency matrix for the graph *G* is a square matrix denoted as *A*(*G*) = *Adj*(*G*) = [*a*_*kl*_], in which [*a*_*kl*_] = 1 when two vertices *v*_*k*_ and *v*_*l*_ are adjacent otherwise it defines to be as zero, mathematically it can be formulated as $A\left(G\right)=\left\{\begin{array}{cc} 1\; & \text{if}v_{k}∼v_{l},\\ 0\; & \text{elsewhere}. \end{array}\right.$ . The adjacency eigenvalues of *G* labelled as $\left(\overset{´}{\gamma_{1}},\overset{´}{\gamma_{2}},\ldots ,\overset{´}{\gamma_{z}}\right)$ are the eigenvalues of *A*(*G*). With the help of these eigenvalues we define the spectrum of related graph *G* (Cvetković et al., 2009) and then concluded their energy.

The graph energy is one of the very few mathematical ideas which are chemically motivated into the modern subject of mathematics. Additionally, Graph theory encompasses various invariants that are significant for understanding the properties of a graph. Among these, the energy and spectral radius hold particular importance. Furthermore, the concept of graph energy, denoted as *ɛ*(*G*), was initially introduced by Gutman in 1978 (Gutman, 1978). However, Initially, this notion was met with skepticism and was explored by only a limited group of scientists due to its unconventional nature. Nevertheless, it wasn’t until the year 2000 that mathematicians truly embraced this concept. At present, the idea of graph energy has gained substantial recognition due to its wide-ranging applications across diverse industries. Consequently, there is a surge of interest in this field, leading to the constant emergence of graph energy and fundamental algebraic identities. Notably, graph energy captures the graphical characteristic, while the spectral radius of a graph represents the largest absolute eigenvalue among all the eigenvalues of its adjacency matrix, denoted by the notation *℘*(*G*). Moreover, these mathematical tools have found several applications in algebraic graph theory (Zhang et al., 2022). Both of these tools, i.e., the energy and spectral radius, play pivotal roles in comprehending the structural properties and behavior of graphs across various disciplines. They offer valuable insights into a graph’s connectivity, stability, expansion, and spread dynamics, making them indispensable for the analysis and characterization of graphs in different contexts.

There is a considerable and visible connection between chemistry and graph theory. Specifically in graph theory, the degree of a molecular graph vertex corresponds to the valency of an atom. Moreover, A variety of topological indices have been built on the product of the degrees *d*_*k*_ and *d*_*l*_ of the terminal vertices *k* and *l* of the edge (chemical bond) *kl*, which has attracted the interest of mathematical chemists. In recent decades, topological indices have undergone extensive research in a number of fields, including mathematics (Gutman and Polansky, 2012; Janezic et al., 2015), physics (Labanowski et al., 1991), biology (Bajorath and Bajorath, 2011). Notably, they have found applications especially in chemical disciplines (Klein et al., 1992; Trinajstić and Nikolić, 2000), such as chemical documentation, isomer discrimination, study of molecular complexity, and other related fields like *QSAR* and *QSPR*, drug design, database choice, etc. A topological index (Gutman, 2013) is a numerical value intrinsically tied to a graph, serving as a fundamental characterizer of the graph’s topology while retaining its consistency through graph transformations. Within the realm of chemical graph theory, degree-based topological indices assume a paramount role and hold immense significant. Topological indices and graph invariants based on vertex degree and the distance between vertices are commonly serve as indispensable tools in characterizing molecular graphs. In the work of Gao et al. (2016a); Gao et al. (2016b) several noteworthy findings on topological indices of chemical graphs have emerged. These techniques establish a profound link between a molecule’s structure attributes and its properties, thereby enabling the predictions of biological activity for chemical compounds, and contributing to the development of various chemical applications. Among these indices, Wiener index, the pioneer and extensively researched topological index, has accumulate significant attention in terms of both theoretical standpoint and practical applications. Similarly, the Zagreb indices, which are degree-based topological indices, have been the subject of extensive exploration and were originally introduced by Gutman and Trinajsti $\overset{´}{c}$ (Gutman and Trinajstić, 1972). These indices provide valuable insights into the graph’s overall structure and the characteristics of its vertices. Specifically, the first Zagreb index, denoted as *ZI*_1_(*G*), is defined as the sum of the squares of the vertex degrees within a molecular graph. On the other hand, the second Zagreb index *ZI*_2_(*G*) corresponds to the sum of the product of the vertex degrees of pairs of adjacent vertices. In a mathematical approach, this relationship is expressed as follow: $ZI_{1}\left(G\right)=\sum_{v\in V\left(G\right)}d_{G}^{2}\left(v\right)=\sum_{kl\in E\left(G\right)}\left(d_{G}\left(k\right)+d_{G}\left(l\right)\right)$ and is *ZI*_2_(*G*) = *∑*_*kl*∈*E*(*G*)_(*d*_*G*_(*k*)*d*_*G*_(*l*)). Moving on, M.O. Albertson (Albertson, 1997) introduced Albertson matrix, which contains the entries ∣*d*_*G*_(*k*) − *d*_*G*_(*l*)∣ when two vertices *v*_*k*_ and *v*_*l*_ are adjacent (*i*.*e*., *v*_*k*_ ∼ *v*_*l*_) and zeroes elsewhere. It is occasionally famed as the Albertson Index (Gutman et al., 2005) and referred to as the third zagreb index in a recent work (Fath-Tabar, 2011). The topological indicator known as the Albertson index serves to describe the structural characteristic of molecule, particulary in the context of quantitative structure activity relationship (*QSAR*) research in chemistry. *QSAR* is a technique employed for predicting a chemical compound’s biological activity or characteristic based on its molecular structure. In essence, it assesses the connectedness, interconnectedness, and robustness of a graph. It also gauges the effectiveness of information or signal transmission within the network and the degree of connectedness between vertices. It’s worth noting that a graph with a higher index value tends to be more robust and resilient.

The significance of energy and spectral radii of a graph extends across diverse fields. In the realm of social network analysis, energy serves as a crucial metric for measuring network stability, while spectral radius highlights influential nodes within the network. Likewise, in the domain of electrical circuit analysis, graphs play a pivotal role in illustrating circuit components and their interconnections. The energy of a graph becomes a meaningful measure, reflecting the total energy or power dissipation within an electrical circuit, while spectral radius aids stability analysis and the determination of maximum gain or amplification within feedback systems. Furthermore, the spectral radius determines visual patterns in the realm of image processing while energy gauges pixel cohesiveness. Beyond this, the energy of a graph finds utility in epidemiology and disease spread modeling, where it can illustrate the potential for disease transmission between individuals. A lower energy value may indicate a situation that is less contagious, providing valuable insights for disease control strategies. On the other hand, the spectral radius plays a key role in the calculation of the basic reproduction number (*R*_0_) in epidemic models, with a larger spectral radius suggesting a higher potential for an outbreak. In addition to these applications, recommender systems utilize energy to gauge user-item compatibility and the spectral radius to identify influential factors. These metrics offer valuable across a spectrum of systems, including social networks, electrical circuits, and epidemiology, enhancing analysis and making predictions more accurate.

The energy and spectral radii of the original graph and those of the splitting and shadow graphs have recently been found to have a substantial relationship. The dimer problem and Huckle′s theory, which serve as examples of how graph spectra are used in statistical physics and chemistry, have also been used in this study to make general findings and emphasise this application. Notably, the utilization of graph spectra in these domains is well-documented, as evidenced by the work of Cvetkovic et al. (1980). Furthermore, Bilal et al. made significant strides in uncovering the significance correlation between the *ISI* energies and the *ISI* spectral radii of the base graph, as well as those of the splitting and shadow graphs, in their recent research (Ahmad et al., 2023). These findings illuminate the structural characteristics of the graph and underscore the interdependence of these measurements. In a complementary vein, another recent study by Ahmad and Munir (2022a) offers noteworthy insights into the strong relation between the *ABC* energies and *ABC* spectral radii of the base graph, as well as those of the splitting and shadow graphs. Bilal et al. in his recent study (Ahmad and Munir, 2022b) unveiled some valueable relationship concerning the randic and reciprocal randic energy and spectral radii of original graph and in correspondence to splitting and shadow graphs. The references (Yu et al., 2004) serve as valuable resources for insights on spectral radii. In (Shao et al., 2021), Shao et al. made a notable discovery regarding the minimum augmented Zagreb energy of trees. Additionally, researchers such as Horn et al. (Horn and Johnson, 1991) and Gatmacher (Gantmacher, 1959) delved into matrix analysis in relation to graph energies, further expanding our understanding of this field. Xavier et al. (2022) described energy of Cartesian product of graph nework. Samir et al. (Vaidya and Popat, 2017a) developed one-splitting and two-shadow graphs of a simple connected graph, revealing that these graphs’ adjacency energies are constant multiples of the energies of the original graph. Expanding on these ideas, Samir et al. (Vaidya and Popat, 2017b) contributed significant findings related to adjacency energies. In Liu et al. (2019) explored the distance and adjacency energy of multiple-level wheel networks, adding another layer of depth to the study of graph energies. The signless Laplacian and Laplacian energies, as well as their spectra, were established by Chu et al. (2020) by means of multi-step wheels. A remarable work on graph energy and its uses was published by I. Gutman et al. in (Li et al., 2012), which included information on more than a hundred different types of graph energies and their applications in various fields. Numerous graph energies have applications in crystallography (Yuge, 2018), as well as in the theory of macromolecules (Pražnikar et al., 2019), biology (Giuliani et al., 2014), protein sequencing (Wu et al., 2015; Yu et al., 2017), air travel problems (Jiang et al., 2016), and spacecraft architecture (Pugliese and Nilchiani, 2017).

In Section 2, we delve into the core concepts centered around the Albertson Estrada (*Alb*) energy and spectral radii of splitting and shadow of regular graphs. Our exploration commences by examining regular graph *G* and formulating general results employing the splitting and shadow graph invariant. Subsequently, we extrapolate from these general findings to obtain specific results that pertain to various regular graph types, including cycle(*C*_*z*_) graphs, complete graphs(*K*_*z*_), complete bipartite(*K*_*z*,*z*_) graphs, etc. The *Alb* spectral radius and energy of the splitting graph and shadow graph come under scrutiny in Section 3.

## 2 Preliminaries

**FIGURE 1:**
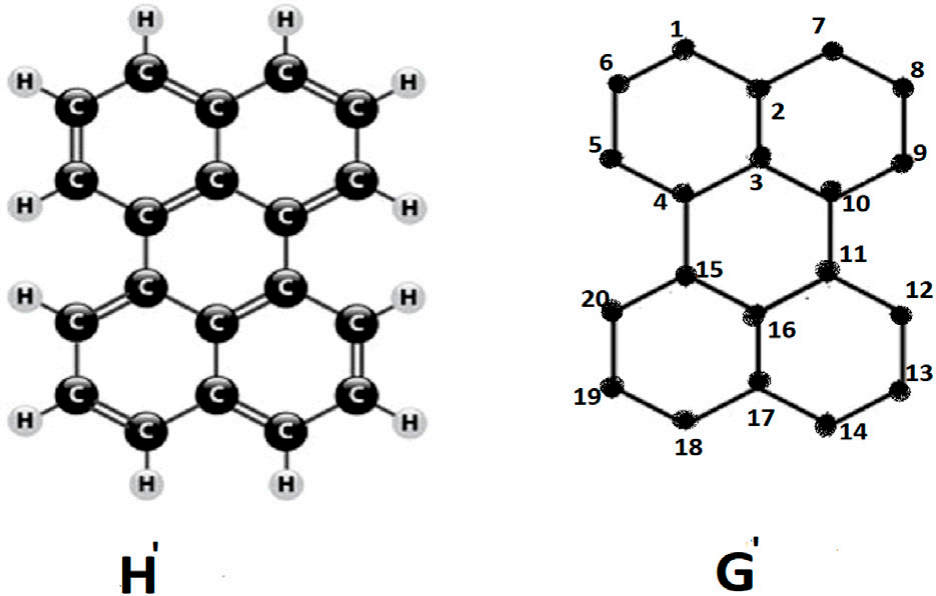
Perylene *H*′ and it’s molecular graph *G*′.

In this section, we will lay out the essential ideas and present pertinent background data related to our key discoveries. Spectral graph theory plays a key role in different applications across different areas ranging from computer science, networking, chemistry, physics, as well as almost all areas of mathematics. The application of spectral graph theory in chemistry is highly significant, particularly in constructing a connection between graph eigenvalues and the levels of energy of molecular orbital associated with $\overset{´}{\pi }$ -electrons in conjugated hydrocarbons. In the context of the H $\ddot{u}$ ckel Molecular Orbital $\left(\overset{´}{H}MO\right)$ approximation (Zimmerman, 2011), the levels of energy of $\overset{´}{\pi }$ -electrons in conjugated hydrocarbon molecules can be more closely related with the eigenvalues of a chemical graph referred to as the molecular graph. To provide a tangible example, consider the chemical structure of Perylene depicted in Figure 1, which serve as a representative conjugated hydrocarbon with the formula $\overset{´}{H}$ . It consists of a total of 20-carbon atoms, it exhibits a graph with *z* = 20.

Perylene, *H*′, is an example of a conjugated hydrocarbon, characterized by its carbon atom structure illustrate as chemical graph *G*′ in above figure. The vertices of the graph *G*′ are denoted as 1, 2, … , 20, which represent the carbon atoms in the chemical formula *H*′. In the H $\ddot{u}$ ckel Molecular Orbital $\left(\overset{´}{H}MO\right)$ model, the wave functions of a conjugated hydrocarbon with a specified number of carbon atoms, indicated as ’*z*,’ are represented as a linear combination within an *z*-dimensional space made up of orthogonal basis functions. Simultaneously, Hamiltonian Matrix, which is a square matrix of order *z* by *z*, is defined as:

$$\overset{´}{H_{ij}}=\left\{\begin{array}{cc} \gamma ,\; & \text{If}\;i=j,\\ \kappa ,\; & \text{If there is a chemical bond between atoms}\;i\;\text{and}\;j,\\ 0,\; & \text{If there is no chemical bond between atoms}\;i\;\text{and}\;j, \end{array}\right.$$

where the parameters *γ* and *κ* are constants. As a demonstration, the $\left(\overset{´}{H}MO\right)$ Hamiltonian matrix related to Perylene is:

$$\overset{´}{H}=\left[\begin{array}{cccccccccccccccccccc} \gamma & \kappa & 0 & 0 & 0 & \kappa & 0 & 0 & 0 & 0 & 0 & 0 & 0 & 0 & 0 & 0 & 0 & 0 & 0 & 0\\ \kappa & \gamma & \kappa & 0 & 0 & 0 & \kappa & 0 & 0 & 0 & 0 & 0 & 0 & 0 & 0 & 0 & 0 & 0 & 0 & 0\\ 0 & \kappa & \gamma & \kappa & 0 & 0 & 0 & 0 & 0 & \kappa & 0 & 0 & 0 & 0 & 0 & 0 & 0 & 0 & 0 & 0\\ 0 & 0 & \kappa & \gamma & \kappa & 0 & 0 & 0 & 0 & 0 & 0 & 0 & 0 & 0 & \kappa & 0 & 0 & 0 & 0 & 0\\ 0 & 0 & 0 & \kappa & \gamma & \kappa & 0 & 0 & 0 & 0 & 0 & 0 & 0 & 0 & 0 & 0 & 0 & 0 & 0 & 0\\ \kappa & 0 & 0 & 0 & \kappa & \gamma & 0 & 0 & 0 & 0 & 0 & 0 & 0 & 0 & 0 & 0 & 0 & 0 & 0 & 0\\ 0 & \kappa & 0 & 0 & 0 & 0 & \gamma & \kappa & 0 & 0 & 0 & 0 & 0 & 0 & 0 & 0 & 0 & 0 & 0 & 0\\ 0 & 0 & 0 & 0 & 0 & 0 & \kappa & \gamma & \kappa & 0 & 0 & 0 & 0 & 0 & 0 & 0 & 0 & 0 & 0 & 0\\ 0 & 0 & 0 & 0 & 0 & 0 & 0 & \kappa & \gamma & \kappa & 0 & 0 & 0 & 0 & 0 & 0 & 0 & 0 & 0 & 0\\ 0 & 0 & \kappa & 0 & 0 & 0 & 0 & 0 & \kappa & \gamma & \kappa & 0 & 0 & 0 & 0 & 0 & 0 & 0 & 0 & 0\\ 0 & 0 & 0 & 0 & 0 & 0 & 0 & 0 & 0 & \kappa & \gamma & \kappa & 0 & 0 & 0 & \kappa & 0 & 0 & 0 & 0\\ 0 & 0 & 0 & 0 & 0 & 0 & 0 & 0 & 0 & 0 & \kappa & \gamma & \kappa & 0 & 0 & 0 & 0 & 0 & 0 & 0\\ 0 & 0 & 0 & 0 & 0 & 0 & 0 & 0 & 0 & 0 & 0 & \kappa & \gamma & \kappa & 0 & 0 & 0 & 0 & 0 & 0\\ 0 & 0 & 0 & 0 & 0 & 0 & 0 & 0 & 0 & 0 & 0 & 0 & \kappa & \gamma & 0 & 0 & \kappa & 0 & 0 & 0\\ 0 & 0 & 0 & \kappa & 0 & 0 & 0 & 0 & 0 & 0 & 0 & 0 & 0 & 0 & \gamma & \kappa & 0 & 0 & 0 & \kappa \\ 0 & 0 & 0 & 0 & 0 & 0 & 0 & 0 & 0 & 0 & \kappa & 0 & 0 & 0 & \kappa & \gamma & \kappa & 0 & 0 & 0\\ 0 & 0 & 0 & 0 & 0 & 0 & 0 & 0 & 0 & 0 & 0 & 0 & 0 & \kappa & 0 & \kappa & \gamma & \kappa & 0 & 0\\ 0 & 0 & 0 & 0 & 0 & 0 & 0 & 0 & 0 & 0 & 0 & 0 & 0 & 0 & 0 & 0 & \kappa & \gamma & \kappa & 0\\ 0 & 0 & 0 & 0 & 0 & 0 & 0 & 0 & 0 & 0 & 0 & 0 & 0 & 0 & 0 & 0 & 0 & \kappa & \gamma & \kappa \\ 0 & 0 & 0 & 0 & 0 & 0 & 0 & 0 & 0 & 0 & 0 & 0 & 0 & 0 & \kappa & 0 & 0 & 0 & \kappa & \gamma \end{array}\right].$$

Based on the given example, it is clear that even in the overall scenario within the $\left(\overset{´}{H}MO\right)$ model, it is necessary to address the eigenvalue-eigenvector dilemma of an approximate Hamiltonian matrix. This matrix follows a specific structure expressed as:

$$\overset{´}{H}=\gamma I_{z}+\kappa A\left(G^{\prime }\right)$$ (2.1)

Here, *γ* and *κ* represent specific constants, *I*_*z*_ denotes the *z*-dimensional unit matrix and the adjacency matrix of a specific graph *G*′ is signifies as *A*(*G*′) comprising the *z* vertices, which represents the carbon-atom framework of the underlying conjugated molecule. The energy levels of total $\overset{´}{\pi }$ -electron are determined by the respective eigenvalue of the adjacency matix of *G*′. By virtue of above equation, the energy levels *ɛ*_*k*_ associated with the $\overset{´}{\pi }$ -electrons exhibit a straightforward connection to the eigenvalues *η*_*k*_ of the graph *G*′.

$$\varepsilon_{k}=\gamma +\kappa \;\eta_{k};\;\;\;k=1,2\cdots \;,20.$$ (2.2)

Within the framework of the $\overset{´}{H}MO$ approximation, the collective *E* of all $\overset{´}{\pi }$ -electrons illustrated as $E=\varepsilon_{\overset{´}{\pi }}\left(G\right)=\sum_{k=1}^{z}g_{k}\varepsilon_{k}$ . In this context, the term ’occupation number’ refers to *g*_*k*_, which signifies the quantity associated with the given property. The main point to note is that the number of $\overset{´}{\pi }$ -electrons (denoted by z) in the conjugated hydrocarbons being studied is equal to the sum of individual counts (*g*_1_ + *g*_2_ + ⋯ + *g*_*z*_). Consequently, this implies that $\varepsilon \left(G\right)=\sum_{k=1}^{z}|\eta_{k}|$ .

Let [*Alb*(*G*)] = *x*_*kl*_ referred as *Albertson matrix* of the graph *G* specified in (Albertson, 1997) possessing entries,

$$x_{kl}=\left\{\begin{array}{cc} |d_{k}-d_{l}|,\; & \text{if}\;v_{k}\;\text{and}\;v_{l}\;\text{are adjacent},\\ 0\; & \text{if}\;k=l,\\ 0\; & \text{elsewhere}. \end{array}\right.$$

Here, the degrees of the vertices *v*_*k*_ and *v*_*l*_ are *d*_*k*_ and *d*_*l*_ respectively. Let eigenvalues of the Albertson matrix of the graph *G* are define as $\overset{´}{\xi_{1}},\overset{´}{\xi_{2}},\cdots \overset{´}{\xi_{z}}$ . The *Alb* eigenvalues makes up the *Alb* spectrum of the graph. If distinct eigenvalues $\overset{´}{\xi_{1}},\overset{´}{\xi_{2}},\cdots \overset{´}{\xi_{z}}$ of Albertson of the graph *G* are define with multiplicities $\overset{´}{m_{1}},\overset{´}{m_{2}},\ldots ,\overset{´}{m_{z}}$ , respectively, then

$$spec\left(Alb\right)=\left(\begin{array}{cccc} \overset{´}{\xi_{1}} & \overset{´}{\xi_{2}} & \cdots & \overset{´}{\xi_{z}}\\ \overset{´}{m_{1}} & \overset{´}{m_{2}} & \cdots & \overset{´}{m_{z}} \end{array}\right).$$ (2.3)

With the help of this spectrum, Albertson (*Alb*) energy of a graph (*G*) is defined as

$$Alb\varepsilon \left(G\right)=\overset{z}{\underset{i=1}{\sum }}\left|\begin{array}{c} \overset{´}{\xi_{i}} \end{array}\right|.$$

In Spectral graph theory, spectral radius stand together with graph energy symbolize as *℘Alb*(*G*) and define as:

$$℘Alb\left(G\right)=\max_{i=1}^{z}\left|\begin{array}{c} \overset{´}{\xi_{i}} \end{array}\right|,$$

Where $\overset{´}{\xi_{i}}$ indicated as the eigenvalue of the Albertson matrix ranging from 1 ≤ *i* ≤ *z*. The following definition are crucial to our conclusions.

**Definition 1.1** Splitting graph *Spl*_*s*_(*G*) of a connected graph *G* is obtained by adding new *s* vertices to each vertex *v* of a graph *G*, making sure that every new vertex is connected to every vertex that is adjacent to *v* in the graph *G*.

**FIGURE 2:**
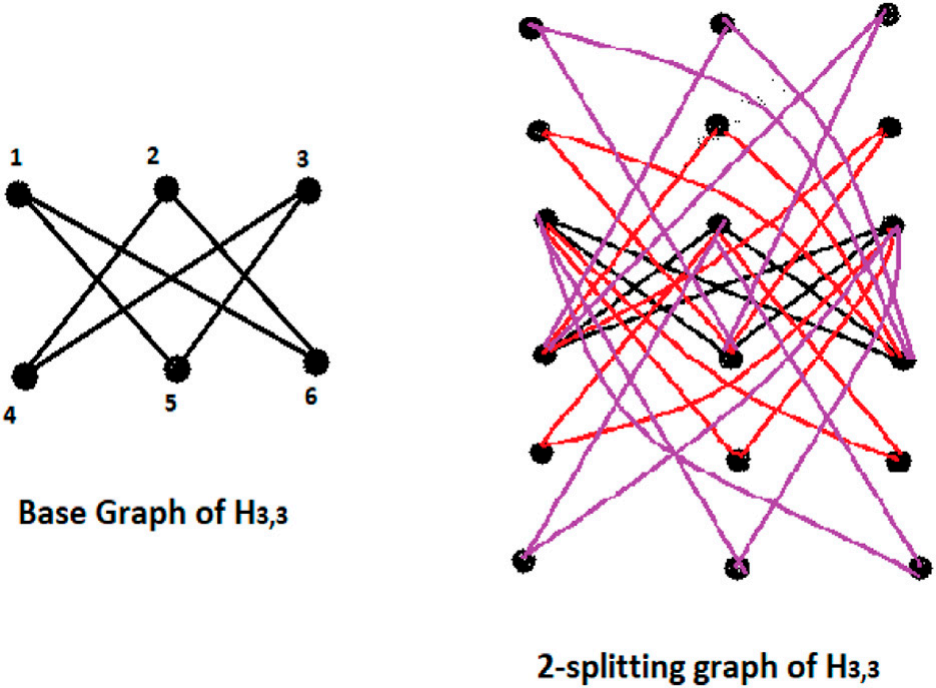
A base graph *H*(3,3) and it’s respective *Spl*_2_(*H*(3,3)).

**FIGURE 3:**
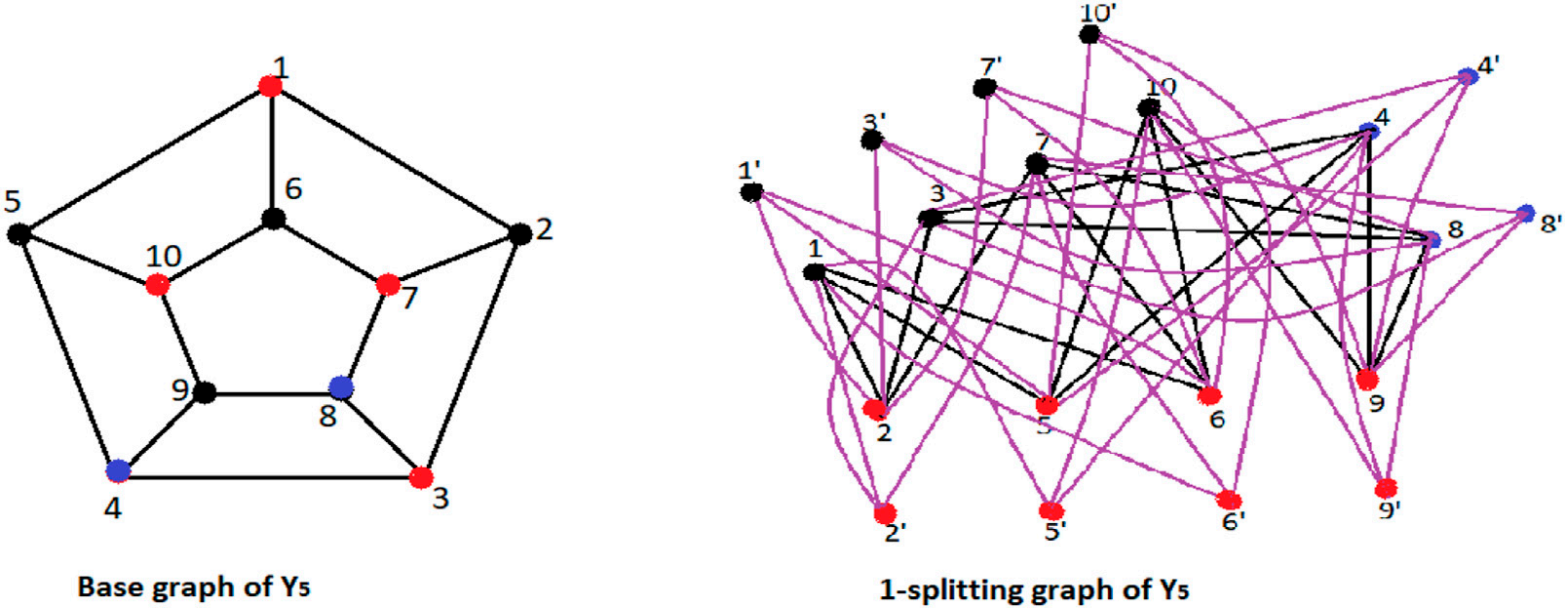
A base graph of (*Y*_5_) and it’s associated *Spl*_1_(*Y*_5_).

Figures 2, 3 should the situation as in Defintion 1.1.

**Definition 1.2** Shadow graph *Sh*_*s*_(*G*) of a connected graph *G* is established by taking *s* copies of *G*, say *G*_1_, *G*_2_, … , *G*_*s*_ then join each vertex *u* in *G*_*i*_ to the neighbours of the corresponding vertex *v* in *G*_*j*_, 1 ≤ *i*, *j* ≤ *s*.

**FIGURE 4:**
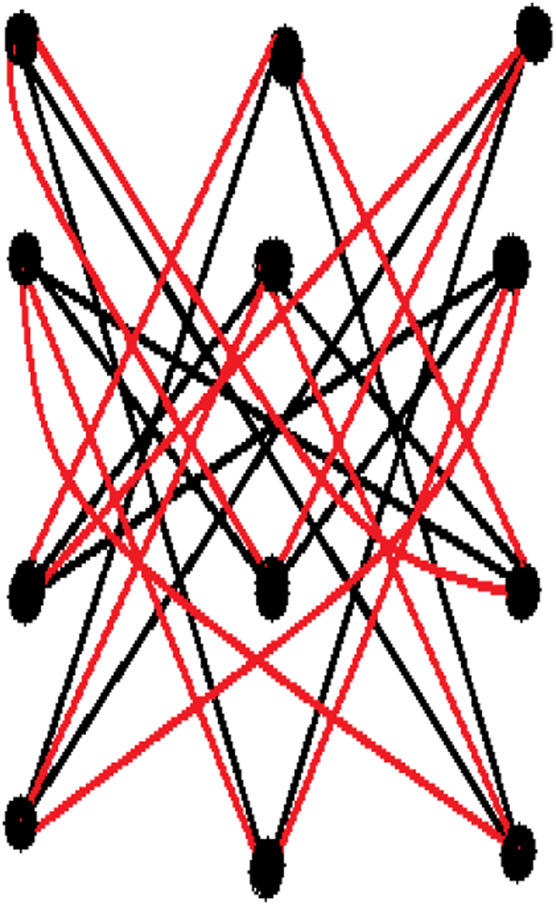
*Sh*_2_(*H*_3,3_), the 2-shadow graph of *H*_3,3_.

Figure 4 should the situation as in definition 1.2. (Neumaier, 1992) Let *AϵR* ^*m*×*n*^, *BϵR* ^*p*×*q*^. Then *A⊗B* is given by

$$A⊗B=\left(\begin{array}{cccc} a_{11}B & . & . & .a_{1n}B\\ . & . & . & .\\ . & . & . & .\\ . & . & . & .\\ a_{m1}B & . & . & .a_{mn}B \end{array}\right).$$

**Proposition 1.1** (Neumaier, 1992) Assuming that *α* is an eigenvalue of *A* and *β* is an eigenvalue of *B*, let’s write *AϵM* ^*m*^, *BϵM* ^*n*^. Then an eigenvalue of *A ⊗ B* is *αβ*.

## 3 Albertson energy and spectral radii of generalized splitting graphs

In this section, we compare the Albertson energies (*Alb*) and spectral radii of generalized splitting and shadow graph of a regular graph *G* with the corresponding Albertson energies (*Alb*) and spectral radii of its original graph. It is essential to emphasize that *G* denotes any *k*-regular graph.

Theorem 1 *If a graph* *G* *is k-regular, then the Albertson energy for the generalized splitting of a regular graph is define as,* $Alb\varepsilon \left(Spl_{s}\left(G\right)\right)=Adj\varepsilon \left(G\right)\left(2ks\sqrt{s}\right)$ .

Proof Let *G* be a *k* regular graph with vertex set *V*(*G*) = {*v*_1_, *v*_2_, *v*_3_, … , *v*_*z*_} and *Spl*_*s*_(*G*) be the splitting graph of graph *G* with vertex set *V*(*Spl*_*s*_(*G*)) = {*v*_11_, *v*_12_, … , *v*_1*z*_, *v*_21_, *v*_22_, … , *v*_2*z*_, … , *v*_*s*1_, *v*_*s*2_, … , *v*_*sz*_} ∪ *V*(*G*). Then *Alb*(*Spl*_*s*_(*G*)) matrix can be written as follows

$$Alb\left(Spl_{s}\left(G\right)\right)={\left[\begin{array}{cccc} {ℵ}_{1} & {ℵ}_{2} & \ldots & {ℵ}_{2}\\ {ℵ}_{2} & {ℵ}_{1} & \ldots & {ℵ}_{1}\\ \vdots & \vdots & \ddots & \vdots \\ {ℵ}_{2} & {ℵ}_{1} & \cdots & {ℵ}_{1} \end{array}\right]}_{s+1},$$

where the matrix *ℵ*_1_ is defined by *ℵ*_1_ = *Alb*(*G*), and the matrix *ℵ*_2_ is defined by *ℵ*_2_ = *ksAdj*(*G*).

$$\begin{array}{ll} Alb\left(Spl_{s}\left(G\right)\right) & ={\left[\begin{array}{cccc} Alb\left(G\right) & ksAdj\left(G\right) & \ldots & ksAdj\left(G\right)\\ ksAdj\left(G\right) & Alb\left(G\right) & \ldots & Alb\left(G\right)\\ \vdots & \vdots & \ddots & \vdots \\ ksAdj\left(G\right) & Alb\left(G\right) & \cdots & Alb\left(G\right) \end{array}\right]}_{s+1}\\ & ={\left[\begin{array}{cccc} Alb\left(G\right)+0Adj\left(G\right) & Alb\left(G\right)+ksAdj\left(G\right) & \ldots & Alb\left(G\right)+ksAdj\left(G\right)\\ Alb\left(G\right)+ksAdj\left(G\right) & Alb\left(G\right)+0Adj\left(G\right) & \ldots & Alb\left(G\right)+0Adj\left(G\right)\\ \vdots & \vdots & \ddots & \vdots \\ Alb\left(G\right)+ksAdj\left(G\right) & Alb\left(G\right)+0Adj\left(G\right) & \cdots & Alb\left(G\right)+0Adj\left(G\right) \end{array}\right]}_{s+1}\\ & ={\left[\begin{array}{ccc} Alb\left(G\right) & \ldots & Alb\left(G\right)\\ \vdots & \ddots & \vdots \\ Alb\left(G\right) & \cdots & Alb\left(G\right) \end{array}\right]}_{s+1}+{\left[\begin{array}{cccc} 0Adj\left(G\right) & ksAdj\left(G\right) & \ldots & ksAdj\left(G\right)\\ ksAdj\left(G\right) & 0Adj\left(G\right) & \ldots & 0Adj\left(G\right)\\ \vdots & \vdots & \ddots & \vdots \\ ksAdj\left(G\right) & 0Adj\left(G\right) & \cdots & 0Adj\left(G\right) \end{array}\right]}_{s+1}\\ & =Alb\left(G\right)⊗{\left[\begin{array}{ccc} 1 & \ldots & 1\\ \vdots & \ddots & \vdots \\ 1 & \cdots & 1 \end{array}\right]}_{s+1}+Adj\left(G\right)⊗{\left[\begin{array}{cccc} 0 & ks & \ldots & ks\\ ks & 0 & \ldots & 0\\ \vdots & \vdots & \ddots & \vdots \\ ks & 0 & \cdots & 0 \end{array}\right]}_{s+1}. \end{array}$$

According to the definition of the Albertson index, *Alb*(*G*) = 0 if and only if a graph *G* is regular.

$$\Rightarrow Alb\left(Spl_{s}\left(G\right)\right)=0+Adj\left(G\right)⊗{\left[\begin{array}{cccc} 0 & ks & \ldots & ks\\ ks & 0 & \ldots & 0\\ \vdots & \vdots & \ddots & \vdots \\ ks & 0 & \cdots & 0 \end{array}\right]}_{s+1}.$$

Let [*B*] = *e*_*kl*_ with their respective entries is define as $e_{kl}={\left[\begin{array}{cccc} 0 & ks & \ldots & ks\\ ks & 0 & \cdots & 0\\ \vdots & \vdots & \ddots & \vdots \\ ks & 0 & \cdots & 0 \end{array}\right]}_{s+1}$ .

To find *Albɛ*(*Spl*_*s*_(*G*)), it is required to obtain all of [*B*]’s eigenvalues. Right now, the [*B*] eigenvalues are being calculated. Because it has rank two this implies that [*B*] has only two eigenvalues that are not zero. The symbol like *α*_2_ and *α*_3_ are stand for the eigenvalues of [*B*]. Therefore, it is evident that

$$\alpha_{2}+\alpha_{3}=tr\left(B\right)=0.$$ (3.1)

Consider [*B* ^2^] = *f*_*kl*_ having entries defines as $f_{kl}={\left[\begin{array}{cccc} s{\left(ks\right)}^{2} & 0 & \ldots & 0\\ 0 & {\left(ks\right)}^{2} & \cdots & {\left(ks\right)}^{2}\\ \vdots & \vdots & \ddots & \vdots \\ 0 & {\left(ks\right)}^{2} & \cdots & {\left(ks\right)}^{2} \end{array}\right]}_{s+1}$ .

Then the trace of above matrix is defines as

$$\alpha_{2}^{2}+\alpha_{3}^{2}=tr\left(B^{2}\right)=s{\left(ks\right)}^{2}+s{\left(ks\right)}^{2}=2k^{2}s^{3}.$$ (3.2)

Equations 3.1, 3.2 when solved yields, $\alpha_{2}=ks\sqrt{s}$ , and $\alpha_{3}=-ks\sqrt{s}$ . The defining equation of [*B*] is denoted by the symbol *Ch*(*B*). Last but not least, we approach at *Ch*(*B*), which can be defined as.

$Ch\left(B\right)=\alpha^{s-1}\left(\alpha -\left(ks\sqrt{s}\right)\right)\left(\alpha +\left(ks\sqrt{s}\right)\right)=\alpha^{s-1}\left(\alpha^{2}-{\left(ks\sqrt{s}\right)}^{2}\right)=0$ .

As a result, we are left with the following spectrum

$$specB=\left(\begin{array}{ccc} 0 & \left(ks\sqrt{s}\right) & -\left(ks\sqrt{s}\right)\\ s-1 & 1 & 1 \end{array}\right).$$ (3.3)

Considering that *Alb*(*Spl*_*s*_(*G*)) = *Adj*(*G*)*⊗B*. Proposition 1.1 is applied, and the result is

$$\begin{array}{ll} Alb\varepsilon \left(Spl_{s}\left(G\right)\right) & =\overset{z}{\underset{i=1}{\sum }}\left|\left.\left(specB\right)\overset{´}{\xi_{i}}\right)\right|\\ & =\overset{z}{\underset{i=1}{\sum }}|\left(\pm ks\sqrt{s}\right)\overset{´}{\xi_{i}}|\\ & =\overset{z}{\underset{i=1}{\sum }}|\overset{´}{\xi_{i}}|\left[ks\sqrt{s}+ks\sqrt{s}\right]\\ & =Adj\varepsilon \left(G\right)\left[2ks\sqrt{s}\right]. \end{array}$$

$$\Rightarrow Alb\varepsilon \left(Spl_{s}\left(G\right)\right)=Adj\varepsilon \left(G\right)\left[2ks\sqrt{s}\right].$$

In view of the above theorem we may interpret this result for some families of regular graphs, i.e., for cycle graph (*C*_*z*_), complete graph (*K*_*z*_), crown graph (*H*_*z*,*z*_), complete bipartite graph (*K*_*z*,*z*_), prism graph (*Y*_*z*_) and hypercube graph (*Q*_*z*_) by assigning values for *k* with respect to that regular graphs.

Proposition 3.1

i) *Alb* *energy of generalized splitting graph of* *C*_*z*_ *is* $Alb\varepsilon \left(Spl_{s}\left(C_{z}\right)\right)=\left(4s\sqrt{s}\right)\left\{\begin{array}{ccc} 4\cot\frac{\pi }{z}, & if & z\equiv 0\left(mod4\right),\\ 4\csc\frac{\pi }{z}, & if & z\equiv 2\left(mod4\right),\\ 2\csc\frac{\pi }{2z}, & if & z\equiv 1\left(mod2\right). \end{array}\right\}$ .
ii) *Alb* *energy of generalized splitting graph of* *K*_*z*_ *is* $Alb\varepsilon \left(Spl_{s}\left(K_{z}\right)\right)=\left[4{\left(z-1\right)}^{2}s\sqrt{s}\right]$ .
iii) *Alb* *energy of generalized splitting graph of* *H*_*z*,*z*_ *is* $Alb\varepsilon \left(Spl_{s}\left(H_{z,z}\right)\right)=\left[8{\left(z-1\right)}^{2}s\sqrt{s}\right]$ .
iv) *Alb* *energy of generalized splitting graph of* *K*_*z*,*z*_ *is* $Alb\varepsilon \left(Spl_{s}\left(K_{z,z}\right)\right)=\left[4z^{2}s\sqrt{s}\right]$ .
v) *Alb* *energy of generalized splitting graph of* *Q*_*z*_ *is* Albε(Spls(Qz))=(2zss)(z+1)2z+1z+12,forz=odd,zzz2,forz=even.

vi) *Alb* *energy of generalized splitting graph of* *Y*_*z*_ *is*

$$Alb\varepsilon \left(Spl_{s}\left(Y_{z}\right)\right)=\overset{z-1}{\underset{j=0}{\sum }}\left(\left|2cos\left(\frac{2\pi j}{z}\right)+1|+|2cos\left(\frac{2\pi j}{z}\right)-1\right|\right)\left(6s\sqrt{s}\right).$$

Proof

i) Cycle graphs, often known as cyclic graphs or just cycles, are a type of mathematical graph that has a closed loop structure. Since cycle graphs (*C*_*z*_) are 2-regular graphs ⇒ *k* = 2, and $Adj\varepsilon \left(C_{z}\right)=\left\{\begin{array}{cc} 4\cot\frac{\pi }{z},\; & \text{if}\;z\equiv 0\left(mod4\right),\\ 4\csc\frac{\pi }{z},\; & \text{if}\;z\equiv 2\left(mod4\right),\\ 2\csc\frac{\pi }{2z},\; & \text{if}\;z\equiv 1\left(mod2\right). \end{array}\right.$ . Hence the result may be obtained by using Theorem 1.
ii) Complete graphs, also referred to as fully connected graphs, are a kind of straightforward undirected graphs in which each pair of distinct nodes is joined by a single edge. Complete graphs are (*z* − 1)-regular graphs ⇒ *k* = (*z* − 1), and *Adjɛ*(*K*_*z*_) = 2(*z* − 1), can be calulated algebrically. Hence, we established result by using Theorem 1.
iii) Crown graph (*H*_*z*,*z*_) on 2z vertices is an undirected graph with two sets of vertices {*u*_1_, *u*_2_, … , *u*_*z*_} and {*v*_1_, *v*_2_, … , *v*_*z*_} with an edge from *u*_*k*_ to *v*_*l*_, whenever *k* ≠ *l*. It is therefore equivalent to the complete bipartite graph (*K*_*z*,*z*_) with horizental edges removed. Crown graphs are (*z* − 1)-regular graphs (*k* = *z* − 1), just like the complete graph while it’s energy defined as *Adjɛ*(*H*_*z*,*z*_) = 4(*z* − 1). Thus, Theorem 1 can be used to reach at the result.
iv) Complete bipartite graphs consist of two sets of vertices say *A* and *B*, in which every vertex in set *A* is linked to every vertex of set *B*. we get *k* = *z* for complete bipartite graphs because it is *z*-regular graphs and their respective energy is defined as *Adjɛ*(*K*_*z*,*z*_) = 2*z*, by applying some basic algebra. Hence, by applying Theorem 1, the result can be achieved.
v) Hypercube graphs, also known as *z*-cube graphs, are a type of mathematical graph that depicts the connectedness among the vertices of a *z*-dimensional hypercube. Just like the complete bipartite, Hypercube graphs are also *z*-regular graphs ⇒ *k* = *z* and Adjε(Qz)=(z+12)z+1z+12 , when z is odd, and Adjε(Qz)=zzz2 , when z is even (Stanley, 2008). Thus, the claim can be achieved by applying Theorem 1.
vi) Prism graphs denoted as *Y*_*z*_ are a type of mathematical graph that are produced by adding additional edges to connect the matching vertices on either side of two cycles. Since the three-dimensional representation of these graphs mimics the shape of a prism, they are known as prism graphs. A *z*-prism graph has 2*z* set of vertices and 3*z* set of edges, and it is equivalent to the generalized Petersen graph *P*(*z*, 1). Prism graphs are 3-regular graphs because it is a part of the cubical graphs, ⇒ *k* = 3 and $Adj\varepsilon \left(Y_{z}\right)=\sum_{j=0}^{z-1}\left(\left|2cos\left(\frac{2\pi j}{z}\right)+1|+|2cos\left(\frac{2\pi j}{z}\right)-1\right|\right)$ in (Gera and Stnic, 2011). Thus, the conclusion can be establish by applying Theorem 1.

Example 3.1 *Consider Latin square graph* (*L*_3_) *which is 6-regular with 9 vetices. Albertson index for Latin square* (*L*_3_) *is defined as*

$$Alb\left(L_{3}\right)={\left[\begin{array}{ccc} 0 & \cdots & 0\\ \vdots & \ddots & \vdots \\ 0 & \cdots & 0 \end{array}\right]}_{9\times 9}.$$

*The matrix for 1-splitting of Latin square* (*L*_3_) *of order 3 is figure out from the following figure:* define as:

$$Spl_{1}\left(L_{3}\right)={\left[\begin{array}{cccccccccccccccccc} 0 & 0 & 0 & 0 & 0 & 0 & 0 & 0 & 0 & 0 & 0 & 0 & 6 & 6 & 6 & 6 & 6 & 6\\ 0 & 0 & 0 & 0 & 0 & 0 & 0 & 0 & 0 & 0 & 0 & 0 & 6 & 6 & 6 & 6 & 6 & 6\\ 0 & 0 & 0 & 0 & 0 & 0 & 0 & 0 & 0 & 0 & 0 & 0 & 6 & 6 & 6 & 6 & 6 & 6\\ 0 & 0 & 0 & 0 & 0 & 0 & 0 & 0 & 0 & 6 & 6 & 6 & 0 & 0 & 0 & 6 & 6 & 6\\ 0 & 0 & 0 & 0 & 0 & 0 & 0 & 0 & 0 & 6 & 6 & 6 & 0 & 0 & 0 & 6 & 6 & 6\\ 0 & 0 & 0 & 0 & 0 & 0 & 0 & 0 & 0 & 6 & 6 & 6 & 0 & 0 & 0 & 6 & 6 & 6\\ 0 & 0 & 0 & 0 & 0 & 0 & 0 & 0 & 0 & 6 & 6 & 6 & 6 & 6 & 6 & 0 & 0 & 0\\ 0 & 0 & 0 & 0 & 0 & 0 & 0 & 0 & 0 & 6 & 6 & 6 & 6 & 6 & 6 & 0 & 0 & 0\\ 0 & 0 & 0 & 0 & 0 & 0 & 0 & 0 & 0 & 6 & 6 & 6 & 6 & 6 & 6 & 0 & 0 & 0\\ 0 & 0 & 0 & 6 & 6 & 6 & 6 & 6 & 6 & 0 & 0 & 0 & 0 & 0 & 0 & 0 & 0 & 0\\ 0 & 0 & 0 & 6 & 6 & 6 & 6 & 6 & 6 & 0 & 0 & 0 & 0 & 0 & 0 & 0 & 0 & 0\\ 0 & 0 & 0 & 6 & 6 & 6 & 6 & 6 & 6 & 0 & 0 & 0 & 0 & 0 & 0 & 0 & 0 & 0\\ 6 & 6 & 6 & 0 & 0 & 0 & 6 & 6 & 6 & 0 & 0 & 0 & 0 & 0 & 0 & 0 & 0 & 0\\ 6 & 6 & 6 & 0 & 0 & 0 & 6 & 6 & 6 & 0 & 0 & 0 & 0 & 0 & 0 & 0 & 0 & 0\\ 6 & 6 & 6 & 0 & 0 & 0 & 6 & 6 & 6 & 0 & 0 & 0 & 0 & 0 & 0 & 0 & 0 & 0\\ 6 & 6 & 6 & 6 & 6 & 6 & 0 & 0 & 0 & 0 & 0 & 0 & 0 & 0 & 0 & 0 & 0 & 0\\ 6 & 6 & 6 & 6 & 6 & 6 & 0 & 0 & 0 & 0 & 0 & 0 & 0 & 0 & 0 & 0 & 0 & 0\\ 6 & 6 & 6 & 6 & 6 & 6 & 0 & 0 & 0 & 0 & 0 & 0 & 0 & 0 & 0 & 0 & 0 & 0 \end{array}\right]}_{18\times 18}.$$ (3.4)

*More precisely,* *Alb*(*Spl*_1_(*L*_3_)) *can be written as follow*

$$Alb\left(Spl_{1}\left(L_{3}\right)\right)={\left[\begin{array}{cc} {ℵ}_{1} & {ℵ}_{2}\\ {ℵ}_{2} & {ℵ}_{1} \end{array}\right]}_{2\times 2}.$$

*Where the matrix* *ℵ*_1_ *is defined by* *ℵ*_1_ = *Alb*(*L*_3_) *and the matrix* *ℵ*_2_ *is defined by* *ℵ*_2_ = 6*Adj*(*L*_3_).

$$\begin{array}{ll} Alb\left(Spl_{1}\left(L_{3}\right)\right) & ={\left[\begin{array}{cc} Alb\left(L_{3}\right) & 6Adj\left(L_{3}\right)\\ 6Adj\left(L_{3}\right) & Alb\left(L_{3}\right) \end{array}\right]}_{2\times 2}\\ & ={\left[\begin{array}{cc} Alb\left(L_{3}\right)+0Adj\left(L_{3}\right) & Alb\left(L_{3}\right)+6Adj\left(L_{3}\right)\\ Alb\left(L_{3}\right)+6Adj\left(L_{3}\right) & Alb\left(L_{3}\right)+0Adj\left(L_{3}\right) \end{array}\right]}_{2\times 2}\\ & ={\left[\begin{array}{cc} Alb\left(L_{3}\right) & Alb\left(L_{3}\right)\\ Alb\left(L_{3}\right) & Alb\left(L_{3}\right) \end{array}\right]}_{2\times 2}+{\left[\begin{array}{cc} 0Adj\left(L_{3}\right) & 6Adj\left(L_{3}\right)\\ 6Adj\left(L_{3}\right) & 0Adj\left(L_{3}\right) \end{array}\right]}_{2\times 2}\\ & =Alb\left(L_{3}\right)⊗\left[\begin{array}{cc} 1 & 1\\ 1 & 1 \end{array}\right]+Adj\left(L_{3}\right)⊗\left[\begin{array}{cc} 0 & 6\\ 6 & 0 \end{array}\right]. \end{array}$$

*Since Latin Square is a regular graph, so by the defintion of the Albertson index* *Alb*(*L*_3_) = 0.

$$\Rightarrow Alb\left(Spl_{s}\left(L_{3}\right)\right)=0+Adj\left(L_{3}\right)⊗\left[\begin{array}{cc} 0 & 6\\ 6 & 0 \end{array}\right].$$

*Let* [*B*] = *c*_*kl*_ *with their respective entries is define as* $e_{kl}=\left[\begin{array}{cc} 0 & 6\\ 6 & 0 \end{array}\right]$ .

*To find* *Albɛ*(*Spl*_1_(*L*_3_))*, it is required to obtain all of* [*B*]*’s eigenvalues. Because it has two rank this implies that* [*B*] *has only two eigenvalues that are not zero. Symbols* *α*_2_ *and* *α*_3_ *stand for the eigenvalues of* [*B*]*. Therefore, it is evident that*

$$\alpha_{2}+\alpha_{3}=tr\left(B\right)=0.$$ (3.5)

*Consider* [*B* ^2^] = *f*_*kl*_ *with their defining entries is* $f_{kl}=\left[\begin{array}{cc} 36 & 0\\ 0 & 36 \end{array}\right]$ *. It’s trace is defined as:*

$$\alpha_{2}^{2}+\alpha_{3}^{2}=tr\left(B^{2}\right)=36+36=72.$$ (3.6)

Equations 3.5, 3.6 *when solved yield the following results* $\alpha_{2}=6\sqrt{2}$ *, and* $\alpha_{3}=-6\sqrt{2}$ . *The characteristic polynomial for* [*B*] *is denoted by* *Ch*(*B*)*, and represented as*. $Ch\left(B\right)=\left(\alpha -6\sqrt{2}\right)\left(\alpha +6\sqrt{2}\right)=\left(\alpha^{2}-{\left(6\sqrt{2}\right)}^{2}\right)=0$ *. As a result, we are left with this spectrum,* $specB=\left(\begin{array}{cc} 6\sqrt{2} & -6\sqrt{2}\\ 1 & 1 \end{array}\right)$ . *In light of the fact that* *Alb*(*Spl*_1_(*L*_3_)) = *Adj*(*L*_3_)*⊗B* *, by applying Proposition (1.1) we get the following outcome:*

$$\begin{array}{ll} Alb\varepsilon \left(Spl_{1}\left(L_{3}\right)\right) & =\overset{2}{\underset{i=1}{\sum }}\left|\left(specB\right)\overset{´}{\xi_{i}}\right|\\ & =\overset{2}{\underset{i=1}{\sum }}|\left(\pm 6\sqrt{2}\right)\overset{´}{\xi_{i}}|\\ & =\overset{2}{\underset{i=1}{\sum }}|\overset{´}{\xi_{i}}\|\pm 6\sqrt{2}|\\ & =Adj\varepsilon \left(L_{3}\right)\left(6\sqrt{2}+6\sqrt{2}\right)\\ & =Adj\varepsilon \left(L_{3}\right)\left(12\sqrt{2}\right), \end{array}$$

*since* $spec\left(Adj\left(L_{3}\right)\right)=\left(\begin{array}{ccc} 6 & -3 & 0\\ 1 & 2 & 6 \end{array}\right)\;\Rightarrow Adj\varepsilon \left(\left(L_{3}\right)\right)=12$

$$\Rightarrow Alb\varepsilon \left(Spl_{1}\left(L_{3}\right)\right)=144\sqrt{2}.$$

***Another approach:*** *Spectrum of* $Alb\left(Spl_{1}\left(L_{3}\right)\right)$ *is easily observed by a direct computation of matrix* *Alb*(*spl*_1_(*L*_3_)) *defined in* Equation 3.5 *as:*

$$spec\left(Alb\left(Spl_{1}\left(L_{3}\right)\right)\right.=\left(\begin{array}{ccccc} 0 & -36 & 36 & 18 & -18\\ 12 & 1 & 1 & 2 & 2 \end{array}\right).$$

*Utilizing* $spec\left(Alb\left(Spl_{1}\left(L_{3}\right)\right)\right.$ *we arrive at* $Alb\varepsilon \left(Spl_{1}\left(L_{3}\right)\right)=144\sqrt{2}$ .*We can easily verify this outcome from* Theorem 1.

Theorem 2 *If a graph G is* *k* *-regular graph, then the spectral radius for the generalized splitting of a regualr graph is deifned as,* $℘Alb\left(Spl_{s}\left(G\right)\right)=℘Adj\left(G\right)\left(ks\sqrt{s}\right)$ .

Proof Using same justifications as Formula (3.3) in Theorem 1, we have $spec\left(B\right)=\left(\begin{array}{ccc} 0 & \left(ks\sqrt{s}\right) & -\left(ks\sqrt{s}\right)\\ s-1 & 1 & 1 \end{array}\right)$ . In light of the fact that *Alb*(*Spl*_*s*_(*G*)) = *Adj*(*G*)*⊗B*. By applyning the Proposition (1.1), we get

$$\begin{array}{ll} ℘Alb\left(Spl_{s}\left(G\right)\right) & =\max_{i=1}^{z}\left|\left(specB\right)\overset{´}{\xi_{i}}\right|\\ & =\max_{i=1}^{z}\left|\left(\pm ks\sqrt{s}\right)\overset{´}{\xi_{i}}\right|\\ & =\max_{i=1}^{z}\left|\overset{´}{\xi_{i}}\right|\left(ks\sqrt{s}\right)\\ & =℘Adj\left(G\right)\left(ks\sqrt{s}\right). \end{array}$$

$$\Rightarrow ℘Alb\left(Spl_{s}\left(G\right)\right)=℘Adj\left(G\right)\left(ks\sqrt{s}\right).$$

By assigning values for *k* with respect to specific regular graphs, we can interpret the outcome of the aforementioned theorem for some families of regular graphs, such as the cycle graph (*C*_*z*_), complete graph (*K*_*z*_), crown graph (*H*_*z*,*z*_), complete bipartite graph (*K*_*z*,*z*_), prism graph (*Y*_*z*_), and hypercube graph (*Q*_*z*_) as follows:

Proposition 3.2 *Albertson* (*Alb*) *spectral radii of generalized splitting graph of:*

i) *C*_*z*_ *is* $℘Alb\left(Spl_{s}\left(C_{z}\right)\right)=4s\sqrt{s}$ .
ii) *k*_*z*_ *is* $℘Alb\left(Spl_{s}\left(K_{z}\right)\right)={\left(z-1\right)}^{2}s\sqrt{s}$ .
iii) *H*_*z*,*z*_ *is* $℘Alb\left(Spl_{s}\left(H_{z,z}\right)\right)={\left(z-1\right)}^{2}s\sqrt{s}$ .
iv) *K*_*z*,*z*_ *is* $℘Alb\left(Spl_{s}\left(K_{z,z}\right)\right)=z^{2}s\sqrt{s}$ .
v) *Q*_*z*_ *is* $℘Alb\left(Spl_{s}\left(Q_{z}\right)\right)=z^{2}s\sqrt{s}$ .
vi) *Y*_*z*_ *is* $℘\left.Alb\left(Spl_{s}Y_{z}\right)\right)=9s\sqrt{s}$ .

Proof

i) As the cycle graphs are 2-regular graphs, so we have *k* = 2 and *℘Adj*(*C*_*z*_) = 2. Thus, Theorem 2 can be used to achieve the desired result.
ii) For complete graphs, we have *k* = (*z* − 1) because it is a (*z* − 1)-regular graphs and *℘Adj*(*K*_*z*_) = (*z* − 1). Thus, by adopting Theorem 2, the required result can be produced.
iii) We pursue our argument in the same way as in (*ii*).
iv) Complete bipartite graphs are *z*-regular graphs ⇒ *k* = *z* and *℘Adj*(*K*_*z*,*z*_) = *z*. The Theorem 2 can therefore be used to achieve the desired result.
v) We continue the same argument as in (*iv*).
vi) Since prism graphs are 3-regular graphs ⇒ *k* = 3 and *℘Adj*(*Y*_*z*_) = 3. Hence, Theorem 2 can be used to produce the desired result.

## 4 Remark

Since *Alb*(*G*) = 0 if and only if graph *G* is regular, and the shadow graphs for a regular graph *G* is again a regular graph ⇒ *Alb*(*sh*_*s*_(*G*)) = 0, and hence their respective Albertson (*Alb*) energies and Albertson (*Alb*) spectral radii is zero, i.e., *Albɛ*(*sh*_*s*_(*G*)) = 0 and *℘Alb*(*sh*_*s*_(*G*)) = 0.

## 5 Conclusion

One of the important development in spectral graph theory, which successfully bridging the realm of mathematics and chemistry, is a graph energy theory that stand with spectral radius. These ideas are explored in the literature by several academic works. We must take on the issue of analyzing the spectral radii and energy of larger graphs. We have presented remarkably comprehensive findings by focusing on splitting and shadow graph invariants. Our research demonstrate that spectral radii and energies of larger graphs are multiples of what was seen in the base graph. Such findings provide insightful information about how to understand the resilience of network and the spread of viruses inside them. The main focus of this research work is on the Albertson energies and Albertson spectral radius of the generalized splitting and shadow graphs built on any regular graph *G* in analogy of the classical ideas of graph energy and spectral radius. Finally we conclude that, the new Albertson energies as well as spectral radii of the modified graph are multiple of the respective energy and spectral radii of the original regular graph known as base graph.

It is important to highlight that, a similar strategy can be used to establish a connection between the spectral radii and energy of the splitting and shadow graph created from a regular graph by choosing any alternative topological index. Graph theory encompasses numerous other operation, such as product and joins, complement and dual graphs, union of graphs, and various kinds of graph product, etc. As a future prospect, one could explore the option of employing different graph operations and then compare the spectral radii and energy of the original graph with those of the newly constructed graphs resulting from the aforementioned operation.

## Data Availability

The original contributions presented in the study are included in the article/Supplementary Material, further inquiries can be directed to the corresponding author.
